# Why Arts Matter to People and Families Living With Dementia: Interprofessional Health Humanities Collaboration

**DOI:** 10.1093/geroni/igaa057.1872

**Published:** 2020-12-16

**Authors:** Teri Kennedy

**Affiliations:** The University of Kansas Medical Center, Kansas City, Kansas, United States

## Abstract

This presentation will share examples of arts-based and creative interventions serving people and their families living with dementia representing evidence-based and promising practices in the United States. Such interventions offer effective non-pharmacological approaches to dementia care including use of the visual arts (e.g., drawings, paintings, sculpture) and performing arts (e.g., music, theatre); literature and writing including reminiscence, biographical approaches, and life story work; photography and Photovoice; and dance and movement as intervention modalities. Current evidence will be presented that demonstrates the effectiveness of arts-based interventions as a form of psycho-social and self-care to alleviate the effects of dementia and enhance the quality of life. Recommendations for future research will be discussed. Strategies will be proposed to develop interprofessional health humanities networks between universities, healthcare systems, libraries, museums, and the arts community to collaborate on the creation of arts-based programs in communities currently without the benefit of such programs.

